# Maximum walking speeds obtained using treadmill and overground robot system in persons with post-stroke hemiplegia

**DOI:** 10.1186/1743-0003-9-80

**Published:** 2012-10-11

**Authors:** Carmen E Capó-Lugo, Christopher H Mullens, David A Brown

**Affiliations:** 1 Department of Physical Therapy and Human Movement Sciences, Northwestern University, 645 North Michigan Avenue, Suite 1100, Chicago, Illinois 60611, USA; 2 Interdepartamental Neuroscience Program (NUIN), Northwestern University, Chicago, USA; 3 Department of Physical Medicine and Rehabilitation, Fienberg School of Medicine, Northwestern University, Chicago, USA

**Keywords:** Stroke, Overground walking, KineAssist, Treadmill, Maximum walking speed

## Abstract

**Background:**

Previous studies demonstrated that stroke survivors have a limited capacity to increase their walking speeds beyond their self-selected maximum walking speed (SMWS). The purpose of this study was to determine the capacity of stroke survivors to reach faster speeds than their SMWS while walking on a treadmill belt or while being pushed by a robotic system (i.e. “push mode”).

**Methods:**

Eighteen chronic stroke survivors with hemiplegia were involved in the study. We calculated their self-selected comfortable walking speed (SCWS) and SMWS overground using a 5-meter walk test (5-MWT). Then, they were exposed to walking at increased speeds, on a treadmill and while in “push mode” in an overground robotic device, the KineAssist, until they were tested at a speed that they could not sustain without losing balance. We recorded the time and number of steps during each trial and calculated gait speed, average cadence and average step length.

**Results:**

Maximum walking speed in the “push mode” was 13% higher than the maximum walking speed on the treadmill and both were higher (“push mode”: 61%; treadmill: 40%) than the maximum walking speed overground. Subjects achieved these faster speeds by initially increasing both step length and cadence and, once individuals stopped increasing their step length, by only increasing cadence.

**Conclusions:**

With post-stroke hemiplegia, individuals are able to walk at faster speeds than their SMWS overground, when provided with a safe environment that provides external forces that requires them to attempt dynamic stability maintenance at higher gait speeds. Therefore, this study suggests the possibility that, given the appropriate conditions, people post-stroke can be trained at higher speeds than previously attempted.

## Background

Individuals post-stroke present an array of changes to neuromuscular system functions such as muscle weakness, impaired proprioception, abnormal muscle activation patterns, and impaired postural control. The different combinations of these and other altered body functions result in limitations in functional mobility, such as reduced gait speed. After stroke, most patients walk at speeds that range from approximately 0.2 m/s to 0.8 m/s [1-4], when asked to walk at a comfortable pace; this comfortable pace will be referred to as self-selected comfortable walking speed (SCWS) from this point forward. These velocities are significantly lower than the SCWS exhibited by age-matched individuals (1.3 m/s to 1.4 m/s) [3-5]. Also, when persons post-stroke were encouraged to walk at their self-selected maximum walking speed (SMWS) they achieved walking speeds from 0.3 m/s to 1.3 m/s [1-3,6]. This suggests that the post-stroke population has limited capability to increase their walking speed to reach higher functional levels because of the real threats to tripping, losing balance, and/or falling, In contrast, non-neurological impaired individuals are able to transition to locomotor states that allow them to achieve very fast speeds rather than fall [7].

Recently, new assessments and interventions targeted at the modulation of gait speed after stroke have incorporated testing and training at fast walking speeds as part of their research and training protocols [4,8-16]. In general, these studies suggest that the analysis of fast walking speed is an important tool to: 1) assess motor impairments [4,11], 2) define and describe biomechanical behaviors at these speeds [8-10,15,16], and 3) challenge patients during training [10-15]. In fact, analysis of gait during fast walking speeds has resulted in the identification of different kinematic and kinetic factors, such as impaired power generation [4]. Additionally, speed-dependent training studies have resulted in significant increases in gait speeds, improved coordination and apparent decreases in step variability [10-16]. However, individuals who have had a stroke do not achieve the functional levels that they had prior to the injury, even after extensive rehabilitation. In the abovementioned research studies, the methods for determining fast walking speed were dependent on safety when individuals were not wearing a harness, or on the participants’ self-determination of walking speed; thus defining the tested speeds as fastest-comfortable or fastest-possible speed. These methods do not allow for a conclusion on the fastest possible speed an individual post-stroke can reach because no study asked individuals to walk beyond their self-selected or perceived maximum walking speed. Therefore, to our knowledge, no study has explicitly tested the limits of the fastest possible gait speeds that can be achieved in persons with post-stroke hemiplegia.

In order to achieve faster walking speeds, non-impaired individuals increase cadence and/or step length, decrease length of stance phase and increase duration of the swing phase, in a symmetric manner [17]. After stroke, individuals show an ability to modulate the abovementioned spatiotemporal parameters [9,18,19], but present with asymmetry between the paretic and non-paretic lower extremities [20-23]. For example, asymmetry consists of prolongation of the duration of stance phase and step length in the non-paretic side, as well as a reduction of the swing time in the non-paretic lower extremity. These deviations have been associated with walking speeds in this population, where walking speed is reduced according to the severity of the deviations [24]. Recently, studies where spatiotemporal parameters have been tested at fast walking speeds suggest that high walking speed is not detrimental to the gait pattern and could potentially reduce gait deviations [16,25]. However, to date no study has tested the limits of the possible speeds that are achievable after stroke and the associated changes to spatiotemporal parameters.

Also, attaining fast walking speeds requires control over the body’s dynamic stability; i.e. the capacity of the neuromuscular system to counteract or prevent a fall during movement [26,27]. In mechanical terms, in order to increase walking speed, rapid changes to the center of mass (CoM) position, CoM velocity, and body momentum are needed [28]. These changes require equally rapid adjustments of the base of support (BoS) in order for one to avoid falling. Researchers have proposed that falls are avoided by keeping the CoM within the feasible stability region (FSR) and margins of stability (MoS) [28]. The FSR is an area within which the neuromuscular system has the capacity to avoid falling by attaining the necessary adjustments. The MoS refers to the boundaries of the FSR beyond which a fall would occur. These variables measured during walking at different speeds, demonstrated that dynamic stability during walking is speed-dependent [26,29,30]. In general, these studies show a reduction of dynamic stability at high walking speeds; likewise, individuals show greater dynamic stability at slow walking speeds.

**Figure 1:**
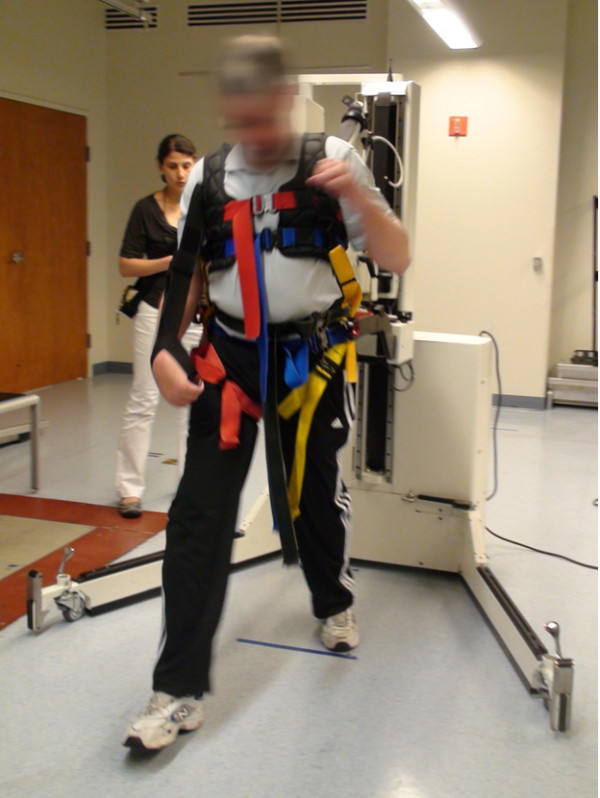
**Overground robotic gait and balance system** (**KineAssist**^**TM**^) **use to provide external forces at the pelvis during walking** (**i.e**. “**push mode**” **walking**).

While a motorized treadmill has been shown to be successful in assisting individuals post-stroke to walk at higher speeds, we sought to test a new robotic system, the KineAssist Walking and Balance Training System (Figure 1), which has the possibility of “pushing” individuals to walk at very high overground speeds and, yet, provide safety in the event of a sudden trip or fall [31,32]. Trips or falls are expected to occur at very high speeds in people post-stroke so this safety mechanism, a pelvic harness system that detects when the pelvis has dropped a certain threshold height, is critical for the study of maximum walking speed capability post-stroke. Also, when the KineAssist is in the “push mode”, it will assist the person to move across the walking surface at very high speeds (up to 2.0 m/s).

With this study, we ask the question: how fast can individuals with post-stroke hemiplegia walk when prompted to the margins of stability at very high speeds? In other words, what are the top speeds that individuals can achieve before they become unstable and lose balance? This assessment of walking beyond SMWS could provide novel insights regarding the capabilities of this population to adapt current neuromuscular mechanisms into more challenging walking tasks. We hypothesized that these participants will be able to walk at higher speeds, compared with overground SMWS, when provided with external forces that assists them to attempt higher gait speeds. To test this hypothesis, we selected two alternate modes for providing assistance with external forces: 1) motorized treadmill walking and, 2) “push mode” walking, generated by an overground robotic device (i.e. the KineAssist, Figure 1). Also, we expected to find increases in step length and cadence according to increments in speed, as well as a top, limiting speed which would consistently result in a loss of balance.

## Methods

### Subjects

Eighteen chronic stroke survivors (age range: 34–81; 59 ± 14 years old) with hemiplegia were recruited from a local database. Subject characteristics are presented in Table 1. Inclusion criteria were: unilateral stroke that resulted in hemiplegia (> 12 months post injury), ability to walk independently without walking aids other than ankle foot orthoses, medically stable (controlled hypertension, no arrhythmia, stable cardiovascular status), and able to provide written informed consent. Exclusion criteria were: history of serious cardiac disease (e.g., myocardial infarction), uncontrolled blood pressure (systolic pressure >140 mmHg, diastolic blood pressure >90 mmHg), presence of cerebellar and brainstem deficits, severe cognitive disorder, uncontrolled respiratory or metabolic disorders, major or acute musculoskeletal problems and body weight greater than 250 pounds (due to robotic device weight restrictions). Also, to help explain the effects of the robotic system walking propulsion, another pilot study was performed in a group of seven healthy, nonimpaired individuals (26 ± 3 years old) with no musculoskeletal, cardiovascular or neurological impairment/pathology that affected their gait performance. Berg Balance and Fugl-Meyer scores were obtained from our laboratory database if the scores were recent (i.e. less than a year old), if scores were not recent, then the investigator performed the evaluations prior to the experimental procedures. These studies were performed at the Department of Physical Therapy and Human Movement Sciences at Northwestern University and written informed consent was obtained according to the policies of Northwestern University Institutional Review Board. Recruitment, clinical testing and experimental procedures were completed by the investigator, who is a licensed physical therapist.

**Table 1:** Characteristics of subjects

**Subject**	**Gender** (**M**/**F**)	**Age** (**years**)	**Paretic Side** (**L**/**R**)	**Months post**-**stroke**	**FM**	**BBS**	**Average SCWS** (**m**/**s**)	**Criteria for Analysis Exclusion**
A	M	55	R	83	25	5	0.86	-
B	M	81	L	58	-	-	0.24	Used treadmill harness for body weight support
C	M	55	L	267	19	45	0.58	-
D*	M	86	L	60	20	38	0.42	Unable to walk without cane during overground and treadmill walking
E	F	57	R	292	20	52	1.07	-
F	F	56	L	154	18	53	0.53	Leaned backward in the robotic device
G	F	39	L	48	15	50	0.75	-
H	F	62	R	59	21	53	0.67	-
I	F	34	L	37	22	50	0.83	-
J	F	44	R	36	20	-	0.65	Refused to be tested at increased speeds on both devices
K	M	69	L	213	19	47	0.83	Refused to be tested at increased speeds while in the treadmill
L	M	51	L	30	17	45	0.86	-
M	M	55	L	84	12	44	0.80	Unable to keep feet within the treadmill belt width
N	F	70	R	254	24	53	0.58	Refused to be tested at increased speeds on both devices
O	F	61	L	219	18	-	0.66	-
P	F	68	L	157	20	53	0.78	Refused to be tested at increased speeds while in the robotic device
Q	M	65	R	126	23	55	0.89	-
R	M	46	R	108	19	46	0.56	Unable to keep feet within the treadmill belt
Mean	9M/9F	59	11L/7R	127	20	46	0.70	-
SD		14		88	3	12	0.20	-

SD, standard deviation; FM, Lower Extremity Fugl Meyer scores; BBS, Berg Balance Test scores; CWS, Self-selected Comfortable Walking Speed; *, subject D was excluded from all data analyses due to an inability to walk without cane during 5-MWT overground.

### Experimental Procedures

After consenting for participation, individuals post-stroke were asked to complete: 1) an overground 5-meter walk test (5-MWT) at self-selected comfortable walking speed (SCWS) and self-selected maximum walking speed (SMWS), 2) a graded 5-MWT on a treadmill belt, and 3) an overground graded 5-MWT while being pushed by a robotic system (“push mode”). The treadmill belt 5-MWT and the “push mode” 5-MWT were presented to each participant in a random order, balanced to assure equal numbers with each order presentation. A computer-program random number generator was used to create a column of randomly-ordered zeros and ones, representing the two conditions to be tested. The number generation was repeated until both conditions generated a list where zeroes and ones were equally represented. Although typical studies use the 10m walk test, we chose to use the shortened variation of the test to minimize fatigue during the more than 20 repeated measures. During each 5-MWT trial the time to walk 5 meters was recoded with a stopwatch and the number of steps was manually counted within the 5 meter distance; steps taken on the start and/or finish line were included. Heart rate and blood pressure were taken immediately before and after each of the 5-MWT tests to ensure that participants had a stable cardiovascular status. See Table 2 for explanation of major variable abbreviations.

**Table 2:** Abbreviations and definitions and abbreviations of the walking speeds measured

**Variable**	**Abbreviation**	**Definition**
Self-selected comfortable walking speed	SCWS	Overground walking speed when the individual was instructed to “*walk at your normal comfortable speed*”
Average self-selected comfortable walking speed	Average SCWS	Comfortable walking speed selected by the participant during an overground 5-MWT average across 3 trials
Self-selected maximum walking speed	SMWS	Overground walking speed when the individual was instructed to “*walk as fast as you safely can*”
Average self-selected maximum walking speed	Average SMWS	Maximum walking speed selected by the participant during an overground 5-MWT average across 3 trials
Top walking speed	TWS	Highest speed that the individual could reach in a single trial overground, on the treadmill and in the robotic device

#### Overground 5-MWT

This 5-MWT test was conducted on an 8-meter walkway. Time and number of steps were recorded from the middle 5 meters and the first and last 1.5 meters were discarded in order to account for acceleration and deceleration phases during gait. Participants were asked to complete the overground 5-MWT by walking without any assistive device at their SCWS and SMWS, three times for each speed. Inability to perform the overground 5-MWT walk without any assistive device resulted in exclusion from the experiment. Then, we calculated the average SCWS and the average SMWS walking speeds.

#### “Push mode” graded 5-MWT

During the “push mode” 5-MWT, participants walked over the same 8-meter walkway as the one described above in the overground 5-MWT. However, in this portion of the study, participants executed the protocol while attached to a robotic device called the KineAssist Gait and Balance Training System™. This robotic device consists of a torso and a pelvis harness attached to a mobile robotic base (Figure 1). This device has been described extensively elsewhere [31,32]. It was used in this experiment for two reasons; to provide a safe walking environment, especially at faster speeds, and to “push” the participant at a variety of fixed walking speeds. Regarding safety, if a person loses balance at any point while they are walking in the device, the device will detect the fall as a drop in the height of their pelvis, will catch the person in the harness system and will simultaneously cease from moving forward. In the “push mode” the mobile base was driven from rest to a fixed, target speed over a short acceleration period (1–2 seconds) so that it pushed the individual to walk at that target speed. The subject’s goal during the task was to keep up with the push and to progress with walking. Subjects were strongly encouraged to maintain normal walking kinematics and were discouraged from leaning back and letting the device carry them forward, any trial where this may have occurred was not used for data analysis. Participants underwent a short familiarization process with the robotic device that consisted of: practice of self-induced falls to establish trust with the safety of the system, free walking to get accustomed to the torso and pelvis harness, and walking in the “push mode” at comfortable speeds. Due to the novelty of the system, we asked participants to decide when they felt comfortable moving in the robotic device and then we concluded the familiarization process.

Participants began the “push mode” graded 5-MWT at their SCWS, as calculated from their overground 5-MWT. After each successful trial, the speed was increased by 0.2 m/s until the participant reached a speed that resulted in 3 consecutive failure trials (e.g. loss of balance resulting in a drop of 3 inches) resulting in loss of balance (the system catches the individual), or refusal to attempt a faster speed. Once 3 consecutive failures were observed, the speed was reduced until 3 successful trials were observed at some consistent speed. This successful speed level was recorded as the top walking speed (TWS) in the “push mode”. A maximum possible test speed of 2 m/s was established *a priori* to ensure a safe environment for the participants and ensure that the device did not become unstable which may happen with excessively high travel speeds. If a participant reached the maximum test speed of 2.0 m/s and no failure was observed then 2 additional trials were attempted and, if successful for three attempts, then 2 m/s was the TWS recorded for that participant.

#### Treadmill graded 5-MWT

During the treadmill 5-MWT, participants were placed in a body harness that was attached to an overhead arch (Biodex Unweighting System) that provided a safety catch in case of a fall. This device did not provide any body weight support during the walking test and any trial where the participant was observed using the system for body weight support was discarded from data analysis. In order to perform the graded 5-MWT on the treadmill (Biodex Gait Trainer 2^TM^) belt a 5-meter distance was measured on the belt and tracked manually. Participants started walking along with the treadmill belt, but the acceleration phase was determined by the treadmill belt, since the test was started once the treadmill reached the desired speed.

The goal for the treadmill graded 5-MWT was similar to the “push mode”. Participants started the test at their calculated SCWS and the speed was increased by 0.2 m/s until the participant attempted a speed where they had 3 consecutive failures or reached the maximum test speed (2.0 m/s). A failure in the treadmill graded 5-MWT was determined by: an inability to maintain an appropriate walking posture (leaning forward, backwards or resting on the harness system), grabbing the handrails, an inability to walk at the set speed resulting in a fall (the system catches the individual), an inability to maintain the base of support (the feet) within the treadmill belt width, and/or refusal to attempt a faster speed. Once 3 consecutive failures were observed at a particular speed, the speed was reduced until 3 successful trials at that speed were accomplished. This final test speed was recorded as the TWS in the treadmill 5-MWT.

#### Characterization of force produced at the pelvis (mini-experiment conducted with nonimpaired, younger participants)

In order to test whether subjects generate the same propulsive forces when freely moving at their desired speed versus when forced during the “push mode”, we characterized the force supplied at the pelvis onto the robot interface by the participants while walking in the robotic device. The KineAssist, without the “push mode”, is driven by a servomechanism that directs the robot position according to the forces detected from the subject at the pelvic mechanism [31,32]. Horizontal force sensed at the pelvis, through load cells located at the pelvic mechanism, was recorded from the robotic device at 100 samples/sec using the EKG data acquisition system. The force supplied at the pelvis was normalized to duration of the steps, and an average across trials for each individual was performed.

Seven healthy, young (less than 40 years old), nonimpaired individuals agreed to walk in the robotic device during “push mode” (the robotic device pushes the individual to walk at a specified speed) and non-“push mode” (the robotic device moves along with the individual at the individual’s selected speed). All trials were conducted on a 14-meter walkway, in which data was only recorded from the middle 10 meters and the first and last 2 meters were discarded. Subjects initially walked in the non-“push mode” and freely chose slow, comfortable, and fast walking speeds, repeated for three trials each. Then, individuals walked in the “push mode” at matched slow, comfortable, and fast walking speeds, presented in random order.

### Data Processing and Analysis

During each trial, the time and number of steps were recorded and the walking speed, average step length, and average cadence were calculated. Speed was determined by dividing the 5-meter distance by the recorded time needed to finish the distance and reported in m/s. Average step length refers to the 5-meter distance divided by the number of steps recorded and reported as m/step. Average cadence was calculated by dividing the number steps recorded by the time needed to complete the 5-meter distance and reported as steps/sec.

Average SCWS refers to the comfortable walking speed selected by the participant during an overground 5-MWT average across 3 trials. Average SMWS refers to the maximum walking speed selected by the participant during an overground 5-MWT average across 3 trials. In both the treadmill walking and “push mode” walking, the comfortable and maximum walking speeds were matched to the closest value of overground SCWS and SMWS. The TWS was defined as the highest speed that the individual could reach in a single trial (see Table 2). Also, the reported average cadence and average step length (not accounting for the asymmetry in hemiparetic step length) during a specific velocity were the values associated with the corresponding speed in each condition.

In order to test the hypothesis that chronic stroke survivors will be able to walk at higher speeds in “push mode” and treadmill walking when compared with SMWS overground, we performed repeated measures ANOVAs with post-hoc Tukey-Kramer analyses (significance selected if adjusted p<0.05). This statistical analysis included testing the differences during each walking condition (overground, treadmill, and “push mode”) for speed (SCWS, SMWS, TWS), and average step length and cadence. Linear regression analyses were used to test for relationships between: 1) average step length or cadence (dependent variables) and walking speed (independent variable) during treadmill and “push mode” walking, and 2) walking speed (on the treadmill and “push mode”) to Fugl-Meyer and Berg Balance Scores. Also, force profiles were graphed in order to visualize the force exerted at the pelvis by healthy participants during the different walking conditions. All statistics were performed using StatView Software (Version 5.0, SAS Institute, Cary, North Carolina) with an α level of 0.05.

## Results

Eighteen people with post-stroke hemiplegia (59 ± 14 years) participated in this study. All eighteen subjects were used in the majority of analyses; however, only nine participants were included in the analyses that required walking at fastest speeds in both the “push mode” and on the treadmill. Even though the harnessed testing systems assured safety at very high speeds, 4 participants refused to attempt faster speeds, 3 participants were unable to walk at increased speeds according to the *a priori* established criteria, and 2 participants were limited by step width which exceeded the width of the treadmill belt (Table 1 and 3).

**Table 3:** **Testing situations during** “**push mode**” **and treadmill walking that resulted in inclusion or exclusion from statistical analyses**

	**Testing Situation**	**Num. of participants**	
		“**Push mode**”	**Treadmill**
**Inclusion**	Able to walk at 2 m/s without losing balance	10	2
	Loss of balance at 2 m/s or less	4	8
**Exclusion**	Loss of balance threshold not reached due to:		
	Participant refused to increase walking speed	3	4
	Technical problems	1	4
	Total number of participants:	18	18

**Figure 2:**
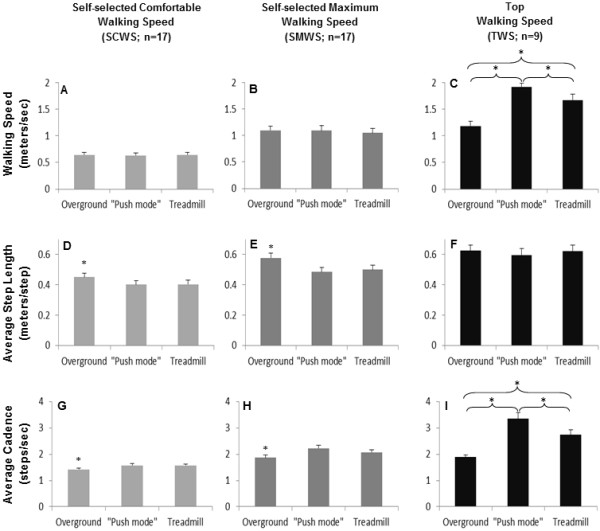
**Comparisons between walking speed** (**A**, **B**, **C**), **average step length** (**D**, **E**, **F**), **and average cadence** (**G**, **H**, **I**) **during overground**, “**push mode**”, **and treadmill walking at three different speeds.** Each column represents mean value ± SEM. Asterisks indicate significant difference (p<0.05) when compared to the other two walking conditions.

Stroke survivors reached significantly faster speeds in the “push mode” (1.92 ± 0.06 m/s) than on the treadmill (1.67 ± 0.11 m/s) (p<0.05) and both were faster than overground (1.19 ± 0.09 m/s) (p<0.05), as seen in Figure 2C. Figure 2C, 2F, and 2I show the walking speed, average step length and average cadence achieved by participants during the top walking speed (TWS). The average step lengths at TWS were similar in the three conditions. However, the average cadence at TMS showed statistically significant differences between conditions, i. e. cadence in the “push mode” (3.53 ± 0.22 steps/s) was significantly faster than in the treadmill (2.74 ± 0.19 steps/s; p<0.05) and both were faster than overground (1.89 ± 0.08 steps/s; p<0.05).

Stroke survivors were able to walk at a grouped average self-selected comfortable speed (SCWS) while overground of 0.67 ± 0.04 m/s. When trials that were selected to match similar walking speeds in the other two walking conditions (“push mode”: 0.66 ± 0.04 m/s; treadmill: 0.67 ± 0.04 m/s; p > 0.05), as shown in Figure 2A, significantly longer average step lengths (Figure 2D) were observed overground when compared to push mode and treadmill walking (overground: 0.45 ± 0.03 m/step; “push mode”: 0.40 ± 0.02 m/step; treadmill: 0.40 ± 0.03 m/step; p<0.05). Also during trials that were selected to matched SCWS, significantly lower average cadences (Figure 2G) were observed overground (1.42 ± 0.05 steps/sec) when compared to “push mode” (1.57 ± 0.07 steps/sec) and treadmill walking (1.58 ± 0.05 steps/sec) (p<0.05).

Likewise, when the self-selected maximum walking speeds (SMWS) overground were chosen to closely match the other two conditions (Figure 2B; overground: 1.10 ± 0.09 m/s; “push mode”: 1.09 ± 0.09 m/s; treadmill: 1.05 ± 0.08 m/s; p > 0.05), the results for average step length and average cadence at SMWS (Figure 2E and Figure 2H, respectively) were similar to those obtained during SCWS.

Linear regression analyses showed that average step length and average cadence were linearly related to walking speed; see Table 4. Average step length in the “push mode” was correlated to walking speed in 9 out of 16 participants while in treadmill walking was correlated in 11 out of 16. On the other hand, cadence in the “push mode” was correlated to walking speed in all 16 participants, while in treadmill walking cadence was correlated to walking speed in 15 out of 16. We did not observe linear relationships between walking speed (on the treadmill and “push mode”) and Berg Balance Scale scores and Lower Extremity Fugl-Meyer.

**Table 4:** **Linear regression analyses results for step length or cadence versus walking speed increments during** “**push mode**” **walking and treadmill walking**

**Subject**^Ŧ^	“**Push Mode**”					**Treadmill**				
	**Step Length**		**Cadence**		***n***	**Step Length**		**Cadence**		***n***
	***M***	***R***^**2**^	***m***	***R***^**2**^		***m***	***R***^**2**^	***m***	***R***^**2**^	
A	0.030	0.059	1.769*	0.886	12	0.027	0.047	1.739*	0.892	11
B	0.509*	0.941	2.188*	0.961	6	-	-	-	-	12
C	0.209*	0.939	0.968*	0.959	13	0.286*	0.804	0.780*	0.675	14
E	0.027	0.049	1.673*	0.857	6	0.186*	0.591	0.886*	0.699	8
F	-	-	-	-	6	0.328*	0.916	0.997*	0.818	6
G	0.157*	0.468	1.138*	0.868	11	0.263	0.624	0986*	0.762	6
H	0.130*	0.773	1.336*	0.948	12	0.296*	0.848	0.724*	0.623	11
I	0.042	0.218	1.886*	0.878	10	0.066	0.195	1.765*	0.837	10
J	0.141*	0.675	1.817*	0.903	10	0.329*	0.832	1.127	0.767	5
K	0.075	0.442	1.316*	0.942	9	0.157	0.464	1.150*	0.785	7
L	0.158*	0.638	0.988*	0.898	9	0.250*	0.846	0.916*	0.910	10
M	0.124*	0.650	1.267*	0.963	10	0.275*	0.900	0.807*	0.882	7
N	0.013	0.006	2.528*	0.867	7	−0.026	0.013	3.216*	0.711	7
O	0.111*	0.800	1.809*	0.960	9	0.130*	0.706	1.743*	0.939	8
P	0.163*	0.862	1.148*	0.863	11	0.178*	0.646	1.221*	0.743	8
Q	0.077	0.385	1.600*	0.888	9	0.149*	0.845	1.204*	0.949	7
R	0.156	0.460	1.452*	0.765	9	0.320*	0.974	0.836*	0.944	8

In terms of balance loss experienced by participants, the number of individuals who experienced a loss of balance was higher in the treadmill and the number of individuals reached the predetermined top speed was higher in the “push mode” (Table 3). Individuals who refused to increase walking speed or were unable to use the systems properly were classified separately and were excluded from the statistical analyses that required use of top walking speeds (TWS) in the “push mode” and on the treadmill (see Table 1 and 3 for more details). Refusal to increase walking speed and inability to use the devices properly impeded the researcher’s ability to determine the individuals’ TWS, previous to a loss of balance. Also, higher number individuals were unable to use the treadmill properly but a similar number of individuals refused to increase their walking speed on both conditions.

**Figure 3:**
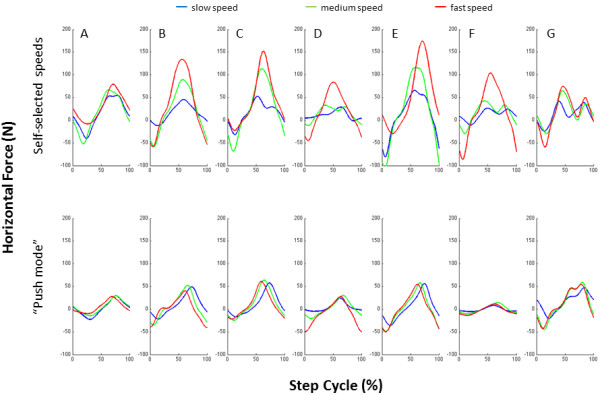
**Force applied at the pelvic mechanism of the robotic device by each healthy** (**n**=**7**) **individual while walking at three different speeds**: **slow** (**blue**), **comfortable** (**green**), **and fast** (**red**) **while in the robotic device**’**s non**-“**push mode**” (**top force profiles**) **and** “**push mode**” (**bottom force profiles**)**.**

In the mini-experiment to characterize the force requirements that were necessary to interact with the KineAssist during the “push mode” versus the non-“push mode”, we observed that the force applied at the pelvis by healthy participants during walking in the robotic device required less force generation during the “push mode” at a given speed than when walking in the robotic device without being pushed (Figure 3). In fact, the force profiles for each individual seem to indicate that in the “push mode” the same amount of force was produced regardless of the walking speed in which they were “pushed” (Figure 3).

## Discussion

This study investigated the capability of individuals with chronic stroke to attain faster walking speeds than their self-selected overground maximum walking speed, when using a treadmill or when pushed overground using a robotic device (“push mode”). The results showed that these individuals were capable of walking significantly faster in treadmill and “push mode” walking compared with their overground self-selected maximum walking speed. Moreover, in many cases they were able to match the top speed limit of the robotic device (2.0 m/sec), indicating that they may have been capable of moving at an even higher velocity. In terms of spatiotemporal parameters, as participants walked under progressively faster conditions, they were able to increase both average step length and average cadence until a maximum average step length was reached and then significant increases in cadence were needed to match speeds. We discuss each of these key results below.

Both conditions, treadmill and “push mode” walking, permitted participants to attain significantly faster walking speeds as compared to their self-selected maximum walking speed overground. This result reaffirms that individuals post-stroke are able to, at least, match their overground walking speeds when walking on a treadmill. In the case of this experiment, when walking in the robotic device; the results are consistent with those that were demonstrated by studies aimed to determine differences between overground and treadmill walking at matched speeds [16,33-39]. It is important to note that in those studies, as in this experiment, walking speeds on the treadmill were determined by the researcher. However, studies where post-stroke individuals self-select their walking speeds on the treadmill revealed that individuals choose speeds that are significantly slower than those self-selected overground [9,40]. Our current study showed that individuals were not only able to match their overground walking speeds, but also had the capability to go beyond those self-selected walking speeds overground.

The ability of individuals to attain faster walking speeds during treadmill and “push mode” walking might be due to common features between the two devices. For instance, both devices provided rhythmically constant external cues at fixed speeds allowing for a more stable and continuous walking pattern than during a self-selected walking speeds. On the treadmill, several experiments in people post-stroke have demonstrated a more symmetrical kinematic gait pattern and decrease step-to-step variability compared to overground walking [33,34]. These more symmetrical walking patterns might be associated with the capability of individuals to reach faster walking speeds. In the robotic device, the only evidence available indicates that no kinematic alterations are observed with functional activities [41,42]. In this way, the device’s predetermined speed requires individuals to choose only those appropriate walking strategies that serve to avoid falling. Our results showed that participants had longer step lengths and slower cadences during overground walking compared to the other two conditions (treadmill and “push mode”) of matched walking speeds. These results have been extensively demonstrated for the treadmill versus overground walking [9,12,33,35].

In addition, participants wore a safety harness during both walking conditions. We propose that, by wearing a safety harness, participants experienced decreased levels of fall-related anxiety (decrease fear or concern of falling) which allowed individuals to appraise their own walking capacities at more challenging and faster speeds. In older individuals, studies suggest that anxiety related to falls is eliminated or reduced by wearing a harness [43]. Also, spatial and temporal parameters are affected not only by physiological constraints, but also by psychological demands [44-46]. Stability-challenging tasks (i.e. walking on elevated or narrow walkways) while in conditions that alter anxiety (i.e. dual tasks or dimmed light) induced decrease walking speeds, shorter steps, decrease cadence, and increase time in double limb support. These studies support a multidimensional model of fear of falling, where fear and anxiety result from an individual’s appraisal of his or her own abilities to maintain balance in combination with other contributors such as falls history [47]. Even though this model has been based on data and experiments performed in older individuals it could be extended to other populations, such as stroke survivors. In the context of this experiment we propose that, by wearing a safety harness, participants experienced decreased levels of fall-related anxiety which allowed individuals to appraise their own walking capacities at more challenging and faster speeds. However, it is important to note that four participants were unwilling to increase their walking speeds in order to avoid experiencing a loss of balance. These participants explicitly stated that they were fearful, apprehensive, or anxious about walking at faster speeds, despite repeated demonstrations that the device was capable of catching them and preventing harm during the experiment. In the case of this group of participants, we think that fear of falling was not reduced, regardless of the use of a harness. In the multidimensional model of fear of falling mentioned above, fear of falling results from an individual’s appraisal of his or her own abilities to maintain balance [47]. This appraisal has three components: physiological, behavioral, and cognitive; all of which could be affected after a stroke. For example, the physiological component is appraised when changes to locomotor control after stroke can negatively impact balance and gait performance. Also, previous falls could lead to the development of the “post-fall” syndrome (i.e. avoidance of activity due to excessive fear of falling) or negatively affecting the cognitive component [47-49]. In our current experiment, none of these components were empirically tested, but our experimental set-up was designed to test the limits of the neurophysiological system. In other words, we asked individuals to increase their walking speed until a loss of balance or a fall was experienced which we interpreted as the fastest speed that the neuromechanical system could accommodate. Thus, we expected individuals to experience some level of fear or concern of falling due to the physiological and cognitive loads imposed to individuals when exposed to this environment in a single session. In summary, the group of participants that were unwilling to attain faster walking speeds represented a subgroup of the post-stroke population in which fear of falling causes an avoidance of participation, whereas in the group of participants that were able to attain faster walking speed, fear of falling was reduced by wearing a harness and demonstration of the ability of the systems to catch people and avoid harm. Also, we propose that the individuals who achieved faster walking speeds were being asked to react to predetermined, stable, and continuous speeds while in provided with a safe environment, in contrast to situations where they are asked to self-select both walking speed and pattern without necessarily providing increased safety assurances.

Participants reached significantly faster speeds during treadmill walking than overground walking, but those speeds were not as fast as those attained in the “push mode” of the robotic device. This result might be influenced by the different biomechanical constraints imposed by each walking environment. Previous investigations that compared treadmill and overground walking have found that, regardless of age, healthy individuals showed minimal changes in spatiotemporal and kinematic gait parameters, but variables such as patterns of muscle activation differ slightly [35-37,50]. In stroke survivors, the evidence suggests even more differences in kinematic and kinetic parameters in treadmill versus overground walking [33,34,38,39]. These studies have shown that during treadmill walking individuals present immediate changes in joint angles, muscle activity, and spatiotemporal parameters that result in more consistent and symmetrical walking patterns. These changes have been attributed mostly to the constraints imposed by the treadmill. For example, when the non-paretic leg is in stance phase, and the treadmill belt moves backwards, the paretic leg is forced to perform a timely swing to maintain the center of mass inside the margins of stability to avoid falling [38]. In this way, not only mechanical changes are observed but also alterations in muscle activity that result in more appropriate walking behaviors [39]. Also, the changes observed in the spatiotemporal parameters seem to be influenced by speed, as demonstrated by several studies [15,16,33]. Our results agree with previous investigations that the mechanical constraints of the treadmill induce mechanical and neuromuscular changes that allow individuals to acquire walking patterns that adjust to faster speeds.

Participants reached their greatest walking speeds during “push mode” and in many cases were able to match the top speed limit of the robotic device (2.0 m/sec), which indicates that they may have been able to walk at even greater speeds. We propose that these results are due to a combination of factors, such as a decreased fear of falling, and biomechanical constraints imposed by the robotic device, among other factors. The first two factors mentioned (fear of falling and speed control) are common to treadmill walking and explained above. On the other hand, the constraints imposed by the robotic device are different from those during treadmill walking. During “push mode” walking, the robotic device moves along with the individual providing a safe overground walking environment, but also allows for control of walking speed. Thus, when the device was set to move at a specified speed, individuals were required to advance their lower extremities, under their own volition and in response to a forward push provided by the device, in order to keep up with the device’s velocity and avoid falling. In other words, the robotic device “pushed” individuals to walk at specified speeds by controlling the velocity and position of the individual’s center of mass. Contrary to the treadmill belt, where the margins of stability are controlled by moving the feet, this pelvic mechanism controls the velocity of the individual’s center of mass. Additionally, the robotic device’s design allowed participants to walk overground which provided congruent sensorimotor information regarding body progression and displacement; contrary to treadmill walking where individuals experience conflicting visual, proprioceptive and vestibular information. Also, the robotic device did not provide assistance with stability since it was developed to allow full degrees of freedom about the hip, pelvis, and trunk so as to challenge individuals to maintain balance [31]. In detail, this robotic device consists of a torso and a pelvis harness attached to a mobile robotic base. The pelvic harness has six degrees of freedom that allow individuals to move in all directions while walking. Both harnesses have a transparent Safety Zone in which the individual can move without any assistance or hindrance from the device. At the boundary of this range the trunk support implements a compliant constraint which catches the patient when he or she loses balance [32]. The combination of these characteristics result in an environment that is safe for attaining fast walking speeds but also similar enough to normal overground walking that the individual is not required to use a different gait strategy than what he or she normally uses for ambulation.

Also, in the mini-experiment with non-impaired subjects reported here, during the “push mode” walking individuals generated less force than at equivalent speeds while in the robotic device without being “pushed”. This reflects decreased force generation to develop forward velocity, allowing subjects to achieve a given velocity with lesser effort. This important finding relates to the determinants of walking speed in post-stroke individuals previously reported. Researchers found a positive correlation between muscle strength and walking speed, both at comfortable and maximum walking speeds [3,4,51-62]. In terms of comfortable walking speed, most studies agreed that the strongest predictor, of walking speed after stroke, was hip and ankle power generation on the paretic leg during the pre- and initial swing phases of the gait cycle [4,51,55,56,59]. In terms of maximum walking speed, the strongest predictors included decreased strength in the hip flexors [4,51,55,57] and ankle plantar flexors [3,4,51,54,55,57,59,61]. Moreover, in the post-stroke population individuals who present a lower functional level, both ankle and hip power generation did not increase with increased voluntary walking speed as they do in controls [4,56]. If the decreased force generation observed in healthy individuals, in this experiment, also occurred in the post-stroke individuals that we studied then we can infer that the robotic device’s “push mode” provided assistance while walking. In other words, the robotic device provided the necessary force to achieve those speeds; thus compensating for individuals’ impairments. This indicated that individuals with impaired power generation would be able to achieve greater walking speeds in the “push mode”. The specific mechanisms by which this assistance occurred in this experiment are unclear, but will be addressed in future studies.

During both the treadmill and “push-mode” conditions, participants increased their average step length and average cadence until a plateau was reached by the average step length and subsequently significant increases in cadence were observed. These results are consistent with previous studies that showed changes in spatiotemporal parameters with increments in speed [9,16,33], implying that post-stroke individuals have the capacity to modify their current walking pattern in order to increase walking speed. Our results suggest that once individuals stopped increasing step length, increments in cadence were the only available modification to the current walking pattern. However, the changes in spatiotemporal parameters observed differ between researchers, as well as the definition of “fast speeds”. For example, during treadmill walking Bayat el al. found increased step length but not cadence [9], Tyrell et al. [16] found longer step lengths, and Brouwer et al. [33] a combination of different kinetic and kinematic factors to attain faster walking speeds [16,33]. Our results showed that participants had longer step lengths and slower cadences during overground walking compared to the other two conditions (treadmill and “push mode”) at matched walking speeds; similar to previously published data [9,33-35]. However, our results also showed that when these individuals were prompted to walk at faster speeds than their maximum overground walking, their step lengths increased to match those of maximum overground walking and subsequently, only increased cadence was observed. These results suggest that once the limit in step length is reached, increments in cadence are the only available modification to the current walking pattern. Therefore, we propose that at fast walking speeds step length became a limiting factor to achieve faster speeds during treadmill and “push mode” walking.

In this experiment, participants were able to walk 3 times faster in the “push mode” and 2.6 times faster during treadmill walking as compared to their overground self-selected comfortable walking pace. Other researchers have done similar comparisons, Tyrell et al. [16] reported walking speeds that were 1.6 times faster than the participants’ self-selected walking pace overground, and Brouwer et al. [33] and Bayat et al. [9] showed speeds 1.2 times faster. We understand that these changes should not be used as direct comparisons because of the inherent differences between each study. Yet, we use this as an indicator of a possible factor that yields the results of our study.

The interpretation of results for this study may be limited by several factors. First, the spatiotemporal parameters measured in this experiment, average step length and cadence, were calculated from the recorded time and steps that participants generated to complete the 5-meter distance. Therefore, the data in this experiment do not provide for individual limb differences or allow for further comparison between stance and swing phases for each leg. However, the average step lengths reported in this experiment are similar to those reported by previous studies [16,33]. Future research that expands and compares the current results with more specific spatiotemporal measurements is needed.

Second, some participants stopped the experiment due to an increased concern or fear of falling. In order to reduce the incidence of this behavior, we provided practice with the safety harness and further verbal encouragement during all trials. For example, once participants expressed their concern they were encouraged to sit on the harness, on both the treadmill and the robotic device, to assure them that they were in a safe environment and reduce their anxiety. In the end, every individual decided if they wanted to continue with the testing procedures. Therefore, we propose that these participants did not reach their maximum walking speeds due to psychological constraints and not because of physical or physiological limitations. The result of psychological stressors on behaviors such as walking is recognized but not widely studied. Thus, it is important that this subgroup is studied and acknowledged during experiments that test individuals at maximum walking speed. We suggest that, in order to study fast walking speeds in this subgroup of post-stroke individuals, attempts at repeated exposure to simplified fast walking tasks where the cognitive load is reduced may result in greater acceptability to move at faster speeds.

Third, equipment constraints possibly limited the ability of participants to reach faster walking speeds. During treadmill walking at fast speeds the width of the treadmill belt was a limiting factor. Participants confined their base of support (step width) within the width of the treadmill in order to avoid tripping and possibly losing balance. For those subjects whose step width seemed to increase with faster walking speeds, this task was troublesome. On the other hand, during overground fast walking speeds in the “push mode”, the robotic device did not maintain a straight line. Consequently, this resulted in longer distances walked in the “push mode” than on the treadmill. These longer walking distances could be due to the individuals’ asymmetry in the walking pattern or asymmetry in force production, among other factors. Future research, evaluating and comparing these two walking environments are needed, as well as careful and detailed studies of the kinetic and kinematic changes at maximal walking speeds.

## Conclusion

The results of this study demonstrated that, after stroke, individuals have the capacity to walk at faster speeds than their overground self-selected maximum walking speed, while walking on a treadmill and in a robotic device. Moreover, in many cases participants were able to match the top speed limit of the robotic device (2.0 m/sec) which indicates that they may have been able to walk at even greater speeds. These changes in walking speed were obtained by initially increasing both step length and cadence. But, once individuals reached the limit of their step length, the only available option was to increase cadence. Therefore, at fast walking speeds step length became a limiting factor to achieve faster speeds during treadmill and “push mode” walking. These results support the growing body of evidence that shows the feasibility and benefits of training this population at high walking speeds.

## Competing interests

DB participates as a consultant with the startup company KineaDesign, LLC, the company that designed and built the KineAssist device. He is listed as an inventor who will potentially receive royalty payments.

## Author’s contributions

CC carried out data collection, analysis and drafted the manuscript. CM assisted in data collection, completed subject recruitment, and helped draft the manuscript. DB participated in the design of the study, statistical analysis, and drafting the manuscript. All authors read and approved the final manuscript.

## Authors’ information

CC was supported in part by a scholarship from the Foundation for Physical Therapy, Inc. and by the Initiative to Maximize Student Development (IMSD) #R25GM079300. DB was supported in part by the U.S. Department of Education, National Institute on Disability and Rehabilitation Research (NIDRR) #H133E070013.
